# Demonstrating the Impact of Cognitive CEO on Firms’ Performance and CSR Activity

**DOI:** 10.3389/fpsyg.2020.00278

**Published:** 2020-02-28

**Authors:** Hui Li, Yong Hang, Syed Ghulam Meran Shah, Aswad Akram, Ilknur Ozturk

**Affiliations:** ^1^Business School, Hohai University, Nanjing, China; ^2^College of Economics and Management, Jiangsu Maritime Institute, Nanjing, China; ^3^Department of Management Sciences, Government College University, Lahore, Pakistan; ^4^School of Business, Sichuan University, Chengdu, China; ^5^Faculty of Economics and Administrative Sciences, Çağ University, Mersin, Turkey

**Keywords:** corporate governance, entrepreneurship psychology, cognitive CEO, SME performance, CSR activity

## Abstract

One aspect of entrepreneurship psychology also clarifies individual characteristics. Drawing from entrepreneurship psychology, the concept of a cognitive CEO has been formulated using the DAE statistical technique. The study elucidates that cognitive CEOs not only boost SME performance but also invigorate CSR activity. Data for listed SMEs for the years 2016–2018 are analyzed through the panel regression technique. Significantly, the study demonstrates that CEO age is positively interlinked with the growth and CSR activity of the firm. Moreover, the empirical underpinning of the results also reveals that state-owned enterprises and firms with high total assets prefer cognitive CEOs, who accelerate the firm’s value and invigorate CSR activity. The number of independent directors is analyzed as a moderator and is concluded to be an intensifier for both SME growth and CSR activity. Finally, 2SLS instrumental panel regression is used to validate the veracity of the empirical results.

## Introduction

The extant literature signifies that excellent corporate governance influences the performance of firms. Significantly, organizational support can mitigate adverse psychological factors among employees (Sarfraz et al., 2019a). However, scant literature has examined the psychological aspects of the upper echelon, specifically among small–medium-sized enterprises (Palmer et al., 2019). Meanwhile, entrepreneurial behavior has been studied using individual attributes (education, age, etc.), showing that these can have a significant impact on the firm’s growth (De Jong et al., 2015). The literature on entrepreneurship psychology has signified through empirical analysis that the role of CEO is pivotal in SMEs and venture capital (Ensley et al., 2002; Lewis, 2015; Miao et al., 2019) while taking strategic decisions.

Specifically, CEO psychology has been demonstrated via a CEO’s specific attributes, which can affect the firm’s performance asymmetrically (Kim et al., 2016; Ou et al., 2018; Park et al., 2018). Certainly, CEO attributes affect different aspects of an organization that are interlinked with the firm’s growth and sustainability. In this regard, analyses have been performed on the impact of CEO attributes on a firm’s cash holdings, innovation, corporate social responsibility, and earnings management (Francis et al., 2008; Orens and Reheul, 2013; Chen et al., 2014; Zhang et al., 2017; García-Sánchez and Martínez-Ferrero, 2019).

CEO characteristics illustrate the innovative capacity of the firm (De Visser and Faems, 2015). Extant studies have found that CEO cognitive style is interlinked with innovative capacity, but the present study contributes new insights by formulating the concept of a cognitive CEO and analyzing its effects on the performance and CSR activity of a firm.

The social cognitive theory emphasizes the individual characteristics that can either embellish or blemish performance through environmental factors (Staples and Webster, 2007). Additionally, leaders who adopt cognitive strategies experience emphatic performance enhancement (Torrence and Connelly, 2019). Meanwhile, cognitive psychology emphasizes that cognition synchronizes the brain to proceed properly (Barsalou, 2014), which ultimately assists the individual in making the right decision. A CEO’s cognitive style also illustrates that he/she is a problem solver when confronting uncertainty within the organization. Moreover, an innovative style identifies a strategy that is related to problem-solving (Sadler-Smith and Badger, 1998). Hence, our independent variable, cognitive CEO, has been formulated on the basis of comparative analysis of the intangible assets ratio of an incumbent CEO along with his/her specific characteristics related to knowledge.

Cognition has been considered a significant tool that augments the innovative capabilities of employees (Chen X. et al., 2019), which necessarily escalates a firm’s growth. In emerging countries, innovative capability has been examined under the auspices of the managerial cognition perspective and environmental strategies. Innovative capabilities are weaker where there is excessive governmental control (Yang et al., 2019). We have selected Chinese firms for this empirical analysis because of their distinguishing characteristics. China adopted a market economy over the last few decades, and the corporate governance mechanism is still quite novel. CSRC^1^ has compelled the organizations to have a specific number of independent directors to enhance corporate governance (Wang et al., 2019). Meanwhile, despite excessive governmental interference in Chinese firms and SMEs,^2^ even small–medium enterprises are growing rapidly while contributing to the Chinese GDP (Cui et al., 2019).

The objective of the study is to contemplate whether a CEO with specific characteristics related to experience and knowledge can be conducive to a firm’s growth. Moreover, it considers whether cognitive ability also orientates the CEO toward adopting CSR activity or not. It is also significant to comprehend the role of independent directors under the auspices of a cognitive CEO.

Our study contributes theoretically and empirically. Firstly, we formulate the variable “cognitive CEO” by executing the DAE statistical technique. Secondly, we demonstrate the impact of a cognitive CEO on SME performance. Thirdly, we also contemplate the impact of a cognitive CEO on corporate social responsibly activity. Fourthly, the role of independent directors examined as a moderator of both performance and CSR activity. Last, we execute 2SLS instrumental panel regression, which indicates that our results are authentic and reliable.

## Theoretical Background and Hypothesis Formulation

The extant literature has revealed that corporate governance not only influences a firm’s growth but also affects corporate social responsibility (Bhagat and Bolton, 2019; Zhou, 2019). One study has indicated that corporate social responsibility even influences employee performance in SMEs (Sarfraz et al., 2018a) while, conversely, another emphasizes the moderating role of CSR, which affects project financing (Sarfraz et al., 2018b). However, the CEO is a pivotal figure who can orientate the firm toward adopting CSR measures. The prior literature has identified CEO attributes that have strongly affected both firm performance and CSR disclosure (García-Sánchez and Martínez-Ferrero, 2019; Hegde and Mishra, 2019). The psychological factors that are related to CEO personality have also been analyzed, revealing their strong impact on organizational risk (Benischke et al., 2019).

An enormous body of literature exists on how managerial cognition relates to environmental strategies, but few studies have been found that signify how the evolution of managerial cognition interlinks with environmental strategies (Yang et al., 2019). Moreover, a study has demonstrated that the intensity of motivation also invigorates cognitive capability (Shepherd and Patzelt, 2018), which ultimately assists individuals in taking drastic steps under unpredictable circumstances. Additionally, it has been witnessed that cognitive behavior under the umbrella of the social aspect does influence strategic decisions (Bromiley and Rau, 2016), as it compels the upper echelon to work diligently for the firm’s growth.

More precisely, prior research on CEOs can be segregated into three categories. First, firm growth and CEO personality in terms of the five personality aspects (conscientiousness, emotional stability, agreeableness, extraversion, and openness) have been examined (Peterson et al., 2003; Nadkarni and Herrmann, 2010). Second, some studies have emphasized specific CEO attributes, including both positive and negative aspects (self-evaluation, charisma, humility, narcissism, overconfidence, and hubris) (Chatterjee and Hambrick, 2007), that influence different features of the firm asymmetrically. Third, some studies have revealed that CEO values (e.g., collectivism, novelty, self-direction, benevolence, and organizational identification) not only influence the growth of a firm but also its corporate social responsibility. Specifically, a recent study has witnessed that CEO cognition also escalates a firm’s performance even if the firm has entered into a declining phase (Liang et al., 2018). Hence, we can encapsulate the above research to formulate the hypothesis that a cognitive CEO should enhance a firm’s growth.

H1: A cognitive CEOs boosts the firm’s growth.

It is at the CEO’s discretion to either adopt innovative measures or to disclose CSR activities (Davidson et al., 2018). Interestingly, prior studies have found interlinks of firms’ CSR activities with CEO demographics and specific characteristics (Borghesi et al., 2014; Davidson et al., 2018). Further, a recent study on materialistic CEOs has demonstrated that they are less oriented toward corporate social responsibility when compared to non-materialistic CEOs (Davidson et al., 2018). However, our variable “cognitive CEO” is based on variables named “CEO experience, CEO education, number of meetings attended by CEO, and goodwill,” which have been contemplated by some studies and revealed to have a positive relationship with CSR activity (Koehn and Ueng, 2010; Huang, 2013; Golden et al., 2017; Chen W.T. et al., 2019). This guides us to the second hypothesis.

H2: Cognitive CEOs enhance CSR activity.

### Independent Directors as a Moderator for Cognitive CEO and Firm Performance, CSR

Chinese organizations have enhanced their corporate structure via introducing a specific number of independent directors (Wang et al., 2019), who have strengthened the firms’ growth through constant vigilance (Tang et al., 2016). Some studies have found that having a high proportion of independent directors mitigates organizational risk (Li et al., 2017), which ultimately invigorates the firm’s profitability. Meanwhile, the presence of independent directors is also conducive to CSR disclosure (Fernández-Gago et al., 2018). Arguably, independent directors are not only representatives of minority shareholders but are also stakeholders that compel the CEOs to promote CSR activity (García-Sánchez and Martínez-Ferrero, 2018). Therefore, the role of the independent director as a moderator should enhance a firm’s growth and CSR activity emphatically. Thus, the following hypotheses are made:

H3a: Independent directors as a moderator augment the firm’s performance.
H3b: Independent directors as a moderator intensify the CSR activity.

## Data Collection and Measures

We have selected SME data listed on Chinese stock exchanges from 2016 through 2018. Data has been accumulated from CSMAR and WIND following the extant literature (Jiang et al., 2013; Zhang and Qu, 2016; McGuinness et al., 2017). The independent variable, cognitive CEO, has been formulated via analyzing the five variables. Following the extant literature, cognition is defined as the knowledge that has been gained through experience and the senses (Chen X. et al., 2019). Consequently, a CEO can utilize his or her cognition to make the right decision. Therefore, we have selected these variables (CEO experience, CEO compensation, the number of meetings attended by the CEO, goodwill, and intangible assets ratio) that signify his/her cognitive ability. CEO tenure, CEO compensation, goodwill, and the number of meetings attended by the CEO represent how much knowledge, either tacit knowledge or working knowledge, the incumbent CEO will gain, which will ultimately affect the intangible assets of the company. Moreover, motivation is also a vigorous vehicle that boosts hidden capabilities to work with enthusiasm. In this regard, CEO compensation^3^ has been included in formulating the cognitive CEO variable. The cognitive CEO variable has been formulated using the DAE^4^ statistical technique. If the incumbent CEO performs better in terms of output (intangible assets ratio) with the given input (the above four variables), then he/she was deemed a cognitive CEO^5^ and was assigned “1”; otherwise, he/she was assigned “0.” Firstly, we have taken the logarithm of cognitive CEO and have assigned the value “1” if its logarithmic value is greater than the mean value; otherwise, it has been assigned the value “0.” Mathematically, the cognitive CEO has been formulated as follows:

(1) $$C\,C\,E\,O=\sum_{\rho =1}^{k}z_{\rho }\,x_{\rho \,m}/\sum_{\rho =1}^{q}k_{\rho }\,y_{\rho \,m},\text{where}\,\rho =1,\ldots ,n$$

In Eq. 1, there are “*q*” inputs and “*k*” outputs. In this case, our output is the intangible assets ratio, whereas our inputs are CEO experience, CEO compensation, number of meetings attended by the CEO, and goodwill.

Equation 1 emphasizes that all input variables (CEO experience, CEO education, number of meetings attended by the CEO, goodwill) should perform very well, causing a boost in the intangible assets of the firm. Additionally, corporate social responsibility disclosure has been formulated in consideration of the 13 attributes. Though the prior literature (Sial et al., 2018), has formulated corporate social responsibility disclosure measures by emphasizing 11 attributes,^6^ our CSR ratio has been formulated by considering 13 attributes (as presented in Table 1). Mathematically,

(2) $$C\,S\,R\,R_{i,t}=\frac{\sum_{P=1}^{13}Z_{P\,i,t}}{N}\;\text{where}\,Z_{p\,i,t}\in \left\{0,13\right\}$$

**TABLE 1:** Formulation of CSR index.

	Attribute	Measurement
1	Whether the firm has indulged in social donation	Yes = 1, No = 0
2	Whether the firm has been verified by a third-party agency	Yes = 1, No = 0
3	Whether the firm refers to the GRI Sustainability Report Guide	Yes = 1, No = 0
4	Whether the firm discloses the protection of shareholders’ rights	Yes = 1, No = 0
5	Whether the firm discloses the protection of creditors’ rights	Yes = 1, No = 0
6	Whether the firm discloses the protection of employee rights	Yes = 1, No = 0
7	Whether the firm discloses the protection of supplier rights	Yes = 1, No = 0
8	Whether the firm discloses the protection of customers’ and consumers’ rights	Yes = 1, No = 0
9	Whether the firm discloses environmental and sustainable development	Yes = 1, No = 0
10	Whether the firm discloses public relations and social welfare undertakings	Yes = 1, No = 0
11	Whether the firm discloses the construction of a social responsibility system and improvement measures	Yes = 1, No = 0
12	Whether the firm discloses safety production content	Yes = 1, No = 0
13	Whether the firm discloses its shortcomings	Yes = 1, No = 0

where “*Z*_*pi*,*t*_” indicates the different attributes for different listed firms.

In Table 1, 13 attributes have been signified for the formulation of CSSR.

Further, following the extant literature (Jiang et al., 2013; Zhu et al., 2016; Ghulam et al., 2019; Sarfraz et al., 2019b; Shah et al., 2019), we have selected the control variables “EPS” (earnings per share), “AGE” (age of cognitive CEO), “Dual” (CEO having two offices), “SOE” (state-owned enterprise), “Firm Size” (logarithm of number of employees), “LNTA” (logarithm of total assets), “Fage” (firm age), and “Leverage.”

### Empirical Models

The panel regression technique is the preferred method for analyzing longitudinal and cross-sectional data. Through confirmation of the Hausman test, fixed effect panel regression has been selected, which also captures the characteristics of unobservable variable characteristics. Mathematically, the panel regression is expressed as follows:

(3) $$P\,e\,r\,f\,o\,r\,m\,a\,n\,c\,e_{i\,t}=\alpha_{0}+\alpha_{1\,i\,t}\,C\,C\,E\,O_{i\,t}+\alpha_{2\,i\,t}\,D\,u\,a\,l_{i\,t}+\alpha_{3\,i\,t}\,A\,G\,E_{i\,t}$$

$$+\alpha_{4\,i\,t}\,S\,O\,E_{i\,t}+\alpha_{5\,i\,t}\,F\,a\,g\,e_{i\,t}+\alpha_{6\,i\,t}\,F\,i\,r\,m\,s\,i\,z\,e_{i\,t}$$

$$+\alpha_{7\,i\,t}\,l\,n\,T\,A_{i\,t}+\alpha_{8\,i\,t}\,L\,e\,v\,e\,r\,a\,g\,e_{i\,t}+\alpha_{9\,i\,t}\,E\,P\,S_{i\,t}+\epsilon_{i\,t}$$

(4) $$C\,S\,S\,R_{i\,t}=\alpha_{0}+\alpha_{1\,i\,t}\,C\,C\,E\,O_{i\,t}+\alpha_{2\,i\,t}\,D\,u\,a\,l_{i\,t}+\alpha_{3\,i\,t}\,A\,G\,E_{i\,t}$$

$$\;+\alpha_{4\,i\,t}\,S\,O\,E_{i\,t}+\alpha_{5\,i\,t}\,F\,a\,g\,e_{i\,t}+\alpha_{6\,i\,t}\,F\,i\,r\,m\,s\,i\,z\,e_{i\,t}$$

$$+\alpha_{7\,i\,t}\,l\,n\,T\,A_{i\,t}+\alpha_{8\,i\,t}\,L\,e\,v\,e\,r\,a\,g\,e_{i\,t}+\alpha_{9\,i\,t}\,E\,P\,S_{i\,t}+\epsilon_{i\,t}$$

(5) $$P\,e\,r\,f\,o\,r\,m\,a\,n\,c\,e_{i\,t}=\alpha_{0}+\alpha_{1\,i\,t}\,\left(C\,C\,E\,O_{i\,t}\times I\,N\,D\,I\,R_{i\,t}\right)+\alpha_{2\,i\,t}\,D\,u\,a\,l_{i\,t}$$

$$+\alpha_{3\,i\,t}\,A\,G\,E_{i\,t}+\alpha_{4\,i\,t}\,S\,O\,E_{i\,t}+\alpha_{5\,i\,t}\,F\,a\,g\,e_{i\,t}$$

$$+\alpha_{6\,i\,t}\,F\,i\,r\,m\,s\,i\,z\,e_{i\,t}+\alpha_{7\,i\,t}\,l\,n\,T\,A_{i\,t}+\alpha_{8\,i\,t}\,L\,e\,v\,e\,r\,a\,g\,e_{i\,t}$$

$$+\alpha_{9\,i\,t}\,E\,P\,S_{i\,t}+\epsilon_{i\,t}$$

(6) $$C\,S\,R\,R_{i\,t}=\alpha_{0}+\alpha_{1\,i\,t}\,\left(C\,C\,E\,O_{i\,t}\times I\,N\,D\,I\,R_{i\,t}\right)+\alpha_{2\,i\,t}\,D\,u\,a\,l_{i\,t}$$

$$+\alpha_{3\,i\,t}\,A\,G\,E_{i\,t}+\alpha_{4\,i\,t}\,S\,O\,E_{i\,t}+\alpha_{5\,i\,t}\,F\,a\,g\,e_{i\,t}$$

$$+\alpha_{6\,i\,t}\,F\,i\,r\,m\,s\,i\,z\,e_{i\,t}+\alpha_{7\,i\,t}\,l\,n\,T\,A_{i\,t}+\alpha_{8\,i\,t}\,L\,e\,v\,e\,r\,a\,g\,e_{i\,t}$$

$$+\alpha_{9\,i\,t}\,E\,P\,S_{i\,t}+\epsilon_{i\,t}$$

Equations 3 and 5 indicate the effect of a cognitive CEO on a firm’s performance. “ROA” and “ROI” have been endorsed as proxies for measuring the firm’s performance (Lin and Lin, 2019; Shah et al., 2019). Additionally, Eqs 4 and 6 demonstrate the impact of a cognitive CEO on CSR activity. The interaction term (CCEO_it_×INDIR_it_) in Eqs 5 and 6 indicates the effect of independent directors as a moderator of firm performance and CSR activity.

### Empirical Results

In this section, the empirical results of panel regression (see Eqs 3–6) are presented. The authenticity of the results has also been assessed through 2SLS instrumental regression. Since cognition is based on knowledge, we have selected CEO technical education as an instrumental variable.^7^ Table 2 details the descriptive statistics. The variables “CCEO” (cognitive CEO), “Degree” (education), “AGE” (age of CEO), “Dual” (CEO having two offices), and “SOE” (state-owned enterprise) are dummy variables. Moreover, the number of observations is almost the same, although some variables have fewer observations due to missing data.

**TABLE 2:** Descriptive statistics.

Variable	Obs	Mean	SD	Min	Max
CSSR	1603	0.6007006	0.1178094	0.2307692	0.9230769
INDIR	1603	3.771678	1.214877	2	13
EPS	1601	0.3041771	0.8210663	−6.859921	17.53427
ROA	1602	0.0952036	2.735636	−6.776046	108.3657
ROI	1599	0.2689851	0.669638	−0.561306	11.85493
Leverage	1601	0.5408962	0.5428913	0.01561	11.50969
CCEO	1592	0.1011307	0.3015964	0	1
AGE	1592	0.0477387	0.2132798	0	1
LNTA	1601	22.30435	1.438454	14.94164	28.55011
FSZ	1601	7.698642	1.473436	1.609438	11.47645
Dual	1573	0.1862683	0.3894468	0	1
Fage	1603	15.80848	4.084944	0	27
SOE	1603	0.5046787	0.5001341	0	1

*Table 2 gives the descriptive statistics of the different variables. “Fage” represents firm age whereas “FSZ” indicates firm size. The descriptive statistics also witness that all variables have a relatively low standard deviation.*

Table 3 illustrates the correlation between variables. The variable “AGE” has the correlation “0.5462,” whereas all other variables have lower correlation values. Hence, there is no threat of the multicollinearity problem.

**TABLE 3:** Correlation matrix.

	CSSR	ROA	ROI	EPS	LEV	CCEO	AGE	LNTA	LNEMP	Dual	SOE
CSSR	1.000										
ROA	0.0059	1.000									
ROI	0.0021	−0.0048	1.000								
EPS	0.0079	0.3725	0.0326	1.000							
LEV	0.0134	−0.3012	−0.0209	−0.1959	1.000						
CCEO	0.039	0.0632	0.0859	−0.0212	0.032	1.000					
AGE	0.0131	0.0569	0.0042	−0.0235	0.0549	0.5472	1.000				
LNTA	−0.0352	0.0108	−0.0074	0.2364	−0.0200	−0.0420	−0.024	1.000			
FSZ	−0.0662	0.0052	−0.0030	0.1590	−0.0722	−0.0272	−0.028	0.4831	1.000		
Dual	0.0404	−0.0099	−0.0293	−0.0501	0.0339	−0.0067	0.009	−0.0697	−0.0619	1.000	
SOE	0.0676	−0.0133	−0.0093	0.0075	0.0082	0.0120	0.001	0.0751	0.0964	−0.0456	1
Fage	0.1948	0.0326	0.0282	0.0368	0.9020	0.0273	0.005	−0.0391	−0.1303	−0.0523	0.071

*Table 3 signifies that all variables are less correlated except “AGE (CEO age)” (“0.5472”), which is also acceptable for empirical analysis.*

Table 4 reveals that a cognitive CEO (CCEO) boosted the firm’s performance significantly (tenth row and third and fourth columns of Table 3). In the third column of Table 4, the coefficient of ROI is “0.118,” whereas the coefficient of ROA is “0.0435” for a cognitive CEO (CCEO). The results support our first hypothesis (H1). Additionally, the variables “lnTA,” “Fage,” and “AGE” positively boosted firm growth. “SOE” is also positively significant, which indicates that having a cognitive CEO in a state-owned enterprise is highly advantageous for escalating firm growth. Meanwhile, the first row of columns (1) and (2) indicates the coefficient values of the interaction term (cognitive CEO term and independent directors). The coefficient values of CCEO^∗^INDIR (0.0955^∗∗^, 0.436^∗∗∗^, respectively, are greater than the coefficient value of CCEO (0.0435^∗∗^, 0.118^∗∗∗^, respectively), which indicates that the presence of independent directors assists even a cognitive CEO in escalating firm growth significantly. This result satisfies hypothesis H3a.

**TABLE 4:** 2SLS Instrumental regression (cognitive CEO and performance).

	(1)	(2)	(3)	(4)
				
Variables	ROA	ROI	ROI	ROA
CCEO*INDIR	0.0955**	0.436***		
	(0.0982)	(0.147)		
EPS	0.169***	0.0233	0.0230	0.169***
	(0.0118)	(0.0200)	(0.0200)	(0.0118)
Leverage	−0.176***	−0.0174	−0.0233	−0.177***
	(0.0172)	(0.0291)	(0.0292)	(0.0172)
AGE	0.146*	0.382**	0.335**	0.140*
	(0.0849)	(0.152)	(0.137)	(0.0798)
LNTA	0.0157*	0.00684*	0.00538*	0.0156*
	(0.00891)	(0.0151)	(0.0151)	(0.00890)
FSZ	−0.00617	0.00148	0.00179	−0.00609
	(0.00859)	(0.0146)	(0.0146)	(0.00860)
Dual	0.0126	−0.0384	−0.0352	0.0135
	(0.0235)	(0.0400)	(0.0402)	(0.0236)
SOE	0.00709*	0.0172*	0.00895*	0.00637*
	(0.0184)	(0.0311)	(0.0314)	(0.0184)
Fage	0.00413**	0.00382*	0.00374*	0.00351*
	(0.00231)	(0.00395)	(0.00395)	(0.00231)
CCEO			0.118***	0.0435**
			(0.0418)	(0.0242)
Constant	0.409**	0.295	0.298	0.409**
	(0.165)	(0.280)	(0.281)	(0.165)
Observations	1,555	1,551	1,551	1,555
*R*^2^	0.204	0.090	0.203	0.203

Standard errors in parentheses. ***p < 0.01, **p < 0.05, *p < 0.1. Table 4 reveals that “CCEO” (Cognitive CEO) and “CCEO*INDIR” are positively significant for “ROA” and “ROI.” In Table 4, the coefficient values of “CCEO*INDIR” are “0.095**” and “0.436***,” whereas the coefficient values of CCEO are “0.118** and “0.409,” respectively. Through comparison, it has been confirmed that, as a moderator, independent directors boost the firm’s growth strongly. Meanwhile, the variables “Fage” (firm age), “SOE,” “AGE” (CEO age), and “LNTA” (total assets) are positively significant.

Table 5 signifies that a cognitive CEO promotes the disclosure of CSR activity [eleventh row and columns (4) and (5)]. Additionally, the interaction of a CCEO and independent directors enhances the CSR activity [first row and columns (1) and (3)]. Specifically, the coefficient values of CCEO^∗^INDIR (0.0244, 0.0170, and 0.0169) are greater than the coefficient values of CCEO (0.00424 and 0.00601), which argues that the vigilance of independent directors has compelled the cognitive CEO to disclose the CSR activity. Moreover, the variables “SOE,” “AGE,” “LNTA,” and “SOE” boost the CSR activity.

**TABLE 5:** 2SLS Instrumental regression (cognitive CEO and CSR activity).

	(1)	(2)	(3)	(4)	(5)
					
Variables	CSRR	CSRR	CSRR	CSRR	CSRR
CCEO*INDIR	0.0244**	0.0170**	0.0169**		
	(0.0317)	(0.0274)	(0.0275)		
EPS	0.00135	0.00126	−0.000290	−0.000301	0.00130
	(0.00382)	(0.00382)	(0.00366)	(0.00366)	(0.00382)
Leverage	−0.00119	−0.000831			−0.00138
	(0.00555)	(0.00555)			(0.00557)
AGE	0.00784**	0.00919**	0.00902**	0.00675**	0.00522*
	(0.0274)	(0.0286)	(0.0286)	(0.0269)	(0.0258)
LNTA	7.15e-05*	6.51e-05*	0.000649**	0.000626*	4.54e-05*
	(0.00288)	(0.00288)	(0.00286)	(0.00286)	(0.00288)
FSZ	−0.00405	−0.00402	−0.00391	−0.00390	−0.00403
	(0.00278)	(0.00278)	(0.00277)	(0.00277)	(0.00278)
Dual	−0.00994	−0.00974	−0.0106	−0.0105	−0.00970
	(0.00761)	(0.00761)	(0.00761)	(0.00762)	(0.00762)
SOE	0.0149**	0.0153**	0.0141**	0.0144**	0.0140**
	(0.00594)	(0.00592)	(0.00593)	(0.00593)	(0.00595)
Fage	0.00528***	0.00526***	0.00520***	0.00522***	0.00530***
	(0.000747)	(0.000747)	(0.000745)	(0.000744)	(0.000747)
CCEO				0.00424**	0.00601**
				(0.00690)	(0.00783)
Constant	0.540***	0.540***	0.527***	0.527***	0.540***
	(0.0534)	(0.0534)	(0.0531)	(0.0531)	(0.0534)
Observations	1,555	1,555	1,556	1,556	1,555
*R*^2^	0.181	0.181	0.180	0.180	0.181

Standard errors in parentheses. ***p < 0.01, **p < 0.05, *p < 0.1. Table 5 reveals that CCEO and CCEO*INDIR are positively significant for CSR ratio (tenth and first row). Additionally, the variables “AGE,” “Fage,” “LNTA,” and “SOE” are positively significant for CSSR.

## Conclusion

Chinese SMEs contribute more than 60% of Chinese GDP (Huang et al., 2016). Therefore, studying Chinese SMEs is worthwhile and necessary to divulge their secrets. Chinese firms are allegedly under the strict surveillance of the government, but their growth is strong and undeterred. Corporate governance among Chinese firms is novel, but its role is vital for sustainability. However, the role of the CEO is pivotal in making decisions and taking drastic steps under uncertain circumstances. In this regard, entrepreneurship psychology orientates the organizational theorist to contemplate the specific attributes of a CEO that can accelerate a firm’s growth. Further, individual psychological aspects of a CEO also matter in adopting CSR measures, as they signify how much of a philanthropist the CEO is. Additionally, CSR disclosure elucidates whether a CEO is concerned about minority shareholders and stakeholders. The concept of a cognitive CEO is based on the idea of best utilization of knowledge (either tacit knowledge or working knowledge) that can assist him/her in achieving goals. Empirical results have unveiled that cognitive CEOs boost firms’ growth and adoption of CSR. Further, cognitive CEOs perform extremely well under the vigilant surveillance of independent directors. Moreover, older CEOs endorse CSR disclosure and boost the firm’s growth. Finally, firms like state-owned enterprises, mature firms, or firms acquiring large total assets show a positive relationship between performance and CSR disclosure. To summarize, this study recommends that firms should prefer CEOs who are mature, have technical knowledge (either economics, law, or engineering), and have long tenure, as they will boost performance and also disclose CSR activities. Further, this study has also suggested that having a specific number of independent directors will enhance performance through their vigilant surveillance.

### Study Limitations

Although this study has contributed a lot, there are some specific limitations that could be addressed in future study. First, the cognitive CEO has been formulated using specific variables and could be reformulated by incorporating different variables. Second, the impact of a cognitive CEO should be demonstrated for different aspects of organizations (e.g., cash holding, earnings management, etc.). Last, it is recommended that the effectiveness of cognitive CEOs for United States or European firms be analyzed.

## Data Availability Statement

The datasets generated for this study are available on request to the corresponding author.

## Ethics Statement

All study procedures were approved by the Ethics Committee of the Hohai University and informed consent of the participations was implied through survey completion.

## Author Contributions

HL and SS conceived the study and were responsible for the design, and development of the data analysis. HL, YH, and AA were responsible for the data collection and analysis. SS and AA was responsible for the data interpretation. SS wrote the first draft of the manuscript. YH and IO reviewed the manuscript. All authors approved the manuscript.

## Conflict of Interest

The authors declare that the research was conducted in the absence of any commercial or financial relationships that could be construed as a potential conflict of interest.

## Footnotes

1 The Chinese Security Regulatory Council compelled Chinese firms to have a minimum of three independent directors among board members to enhance corporate governance (Liu et al., 2015).

2 According to Zhao et al. (2013), 99% of Chinese chemical firms are SMEs, which means that corporate social responsibility is the main concern for these companies.

3 CEO compensation has a positively significant impact on performance (Jian and Lee, 2015).

4 DAE is a statistical technique used to demonstrate the relative efficiency of separable variables (Demerjian P. et al., 2012).

5 Yuan et al. (2019) and Demerjian P.R. et al. (2012), analyzed the impact of CEO ability and manager ability, formulated on the basis of the DAE technique.

6 A prior study (Yuan et al., 2019) emphasized seven attributes related to reporting.

7 The correlation between instrumental variable (CEO technical education) and cognitive CEO is “0.7863” while with CSSR is “0.0098,” which satisfy the condition of an instrumental variable. The technical education is a dummy variable which equals “1” if the CEO has a degree related to economics, management sciences or law, and “0” otherwise (Ghulam et al., 2019).

## References

[B1] Barsalou L. W. (2014). *Cognitive Psychology: An Overview of Cognitive Scientists.* Hove: Psychology Press.

[B2] Benischke M. H. Martin G. P. Glaser L. (2019). CEO equity risk-bearing and strategic risk-taking: the moderating effect of CEO personality. *Strateg. Manag. J.* 40 153–177. 10.1002/smj.2974

[B3] Bhagat S. Bolton B. (2019). Corporate governance and firm performance: the sequel. *J. Corp. Finance* 58 142–168. 10.1016/j.jcorpfin.2019.04.006

[B4] Borghesi R. Houston J. F. Naranjo A. (2014). Corporate socially responsible investments: CEO altruism, reputation, and shareholder interests. *J. Corp. Finance* 26 164–181. 10.1016/j.jcorpfin.2014.03.008

[B5] Bromiley P. Rau D. (2016). Social, behavioural, and cognitive influences on upper echelons during the strategy process: a literature review. *J. Manag.* 42 174–202. 10.1177/0149206315617240

[B6] Chatterjee A. Hambrick D. C. (2007). It’s all about me: narcissistic chief executive officers and their effects on company strategy and performance. *Adm. Sci Q.* 52 351–386. 10.2189/asqu.52.3.351

[B7] Chen S. S. Ho K. Y. Ho P. H. (2014). CEO overconfidence and long−term performance following R&D increases. *Financial Manag.* 43 245–269. 10.1111/fima.12035

[B8] Chen W. T. Zhou G. S. Zhu X. K. (2019). CEO tenure and corporate social responsibility performance. *J. Bus. Res.* 95 292–302. 10.1016/j.jbusres.2018.08.018

[B9] Chen X. Liu J. Zhang H. Kwan H. K. (2019). Cognitive diversity and innovative work behaviour: the mediating roles of task reflexivity and relationship conflict and the moderating role of perceived support. *J. Occup. Organ. Psychol.* 92 671–694. 10.1111/joop.12259

[B10] Cui Y. Zhang Y. Guo J. Hu H. Meng H. (2019). Top management team knowledge heterogeneity, ownership structure and financial performance: evidence from Chinese IT listed companies. *Technol. Forecasting Soc. Change* 140 14–21. 10.1016/j.techfore.2018.12.008

[B11] De Jong J. P. Parker S. K. Wennekers S. Wu C. H. (2015). Entrepreneurial behaviour in organizations: does job design matter? *Entrep. Theory Pract.* 39 981–995. 10.1111/etap.12084

[B12] De Visser M. Faems D. (2015). Exploration and exploitation within firms: the impact of CEOs’ cognitive style on incremental and radical innovation performance. *Creat. Innov. Manag.* 24 359–372. 10.1111/caim.12137

[B13] Demerjian P. Lev B. McVay S. (2012). Quantifying managerial ability: a new measure and validity tests. *Manag. Sci.* 58 1229–1248. 10.1287/mnsc.1110.1487

[B14] Demerjian P. R. Lev B. Lewis M. F. McVay S. E. (2012). Managerial ability and earnings quality. *Accoun. Rev.* 88 463–498. 10.2308/accr-50318

[B15] Ensley M. D. Pearson A. W. Amason A. C. (2002). Understanding the dynamics of new venture top management teams: cohesion, conflict, and new venture performance. *J. Bus. Ventur.* 17 365–386. 10.1016/s0883-9026(00)00065-3

[B16] Fernández-Gago R. Cabeza-García L. Nieto M. (2018). Independent directors’ background and CSR disclosure. *Corp. Soc. Responsib. Environ. Manag.* 25 991–1001. 10.1002/csr.1515

[B17] Francis J. Huang A. H. Rajgopal S. Zang A. Y. (2008). CEO reputation and earnings quality. *Contemp. Account. Res.* 25 109–147.

[B18] García-Sánchez I. M. Martínez-Ferrero J. (2018). How do independent directors behave with respect to sustainability disclosure? *Corp. Soc. Responsib. Environ. Manag.* 25 609–627. 10.1002/csr.1481

[B19] García-Sánchez I. M. Martínez-Ferrero J. (2019). Chief executive officer ability, corporate social responsibility, and financial performance: the moderating role of the environment. *Busi. Strategy Environ.* 28 542–555.

[B20] Ghulam S. Shah M. Muddassar S. Fareed Z. Ateeq ur Rehman M. Maqbool A. (2019). Whether CEO succession via hierarchical jumps is detrimental or blessing in disguise? Evidence from Chinese listed firms. *Zagreb Int. Rev. Econo. Bus.* 22 23–42.

[B21] Golden J. Sun L. Zhang J. H. (2017). Corporate social responsibility and goodwill impairment. *Account. Public Interest* 18 1–28. 10.2308/apin-51971

[B22] Hegde S. P. Mishra D. R. (2019). Married CEOs and corporate social responsibility. *J. Corp. Finance* 58 226–246. 10.1016/j.jcorpfin.2019.05.003

[B23] Huang S. K. (2013). The impact of CEO characteristics on corporate sustainable development. *Corp. Soc. Responsib. Environ. Manag.* 20 234–244. 10.1002/csr.1295

[B24] Huang W. Boateng A. Newman A. (2016). The capital structure of Chinese listed SMEs: an agency theory perspective. *Small Bus. Econ.* 47 535–550. 10.1007/s11187-016-9729-6

[B25] Jian M. Lee K. W. (2015). CEO compensation and corporate social responsibility. *J. Multinat. Financial Manag.* 29 46–65. 10.1016/j.mulfin.2014.11.004 10276817

[B26] Jiang F. Huang J. Kim K. A. (2013). Appointments of outsiders as CEOs, state-owned enterprises, and firm performance: evidence from China. *Pacific Basin Finance J.* 23 49–64. 10.1016/j.pacfin.2013.01.003

[B27] Kim J. B. Wang Z. Zhang L. (2016). CEO overconfidence and stock price crash risk. *Contemp. Account. Res.* 33 1720–1749. 10.1111/1911-3846.12217

[B28] Koehn D. Ueng J. (2010). Is philanthropy being used by corporate wrongdoers to buy goodwill? *J. Manag. Gov.* 14 1 10.1007/s10997-009-9087-8

[B29] Lewis K. V. (2015). Enacting entrepreneurship and leadership: a longitudinal exploration of gendered identity work. *J. Small Busi. Manag.* 53 662–682. 10.1111/jsbm.12175

[B30] Li T. Munir Q. Karim M. R. A. (2017). The nonlinear relationship between CEO power and capital structure: evidence from China’s listed SMEs. *Int. Rev. Econo, Finance* 47 1–21. 10.1016/j.iref.2016.09.005

[B31] Liang X. Barker V. L. Schepker D. J. (2018). Chief executive cognition, turnaround strategy and turnaround attempts of declining firms. *J. Change Manag.* 18 304–326. 10.1080/14697017.2018.1464046

[B32] Lin H. C. Lin P. C. (2019). The interplay between CEO-TMT exchange level and differentiation: implications for firm competitive behaviours and performance. *J. Bus. Res.* 95 171–181. 10.1016/j.jbusres.2018.10.034

[B33] Liu Y. Miletkov M. K. Wei Z. Yang T. (2015). Board independence and firm performance in China. *J. Corp. Finance* 30 223–244. 10.1016/j.jcorpfin.2014.12.004

[B34] McGuinness P. B. Vieito J. P. Wang M. (2017). The role of board gender and foreign ownership in the CSR performance of Chinese listed firms. *J. Corp. Finance* 42 75–99. 10.1016/j.jcorpfin.2016.11.001

[B35] Miao Q. Eva N. Newman A. Cooper B. (2019). CEO entrepreneurial leadership and performance outcomes of top management teams in entrepreneurial ventures: the mediating effects of psychological safety. *J Small Bus. Manag.* 57 1119–1135. 10.1111/jsbm.12465

[B36] Nadkarni S. Herrmann P. O. L. (2010). CEO personality, strategic flexibility, and firm performance: the case of the Indian business process outsourcing industry. *Acad. Manag. J.* 53 1050–1073. 10.5465/amj.2010.54533196

[B37] Orens R. Reheul A. M. (2013). Do CEO demographics explain cash holdings in SMEs? *Eur. Manag. J.* 31 549–563. 10.1016/j.emj.2013.01.003

[B38] Ou A. Y. Waldman D. A. Peterson S. J. (2018). Do humble CEOs matter? An examination of CEO humility and firm outcomes. *J. Manag.* 44 1147–1173. 10.1177/0149206315604187

[B39] Palmer C. Niemand T. Stöckmann C. Kraus S. Kailer N. (2019). The interplay of entrepreneurial orientation and psychological traits in explaining firm performance. *J. Bus. Res.* 94 183–194. 10.1016/j.jbusres.2017.10.005

[B40] Park J. H. Kim C. Chang Y. K. Lee D. H. Sung Y. D. (2018). CEO hubris and firm performance: exploring the moderating roles of CEO power and board vigilance. *J. Bus. Ethics* 147 919–933. 10.1007/s10551-015-2997-2

[B41] Peterson R. S. Smith D. B. Martorana P. V. Owens P. D. (2003). The impact of chief executive officer personality on top management team dynamics: one mechanism by which leadership affects organizational performance. *J. f Appl. Psychol.* 88:795. 10.1037/0021-9010.88.5.795 14516245

[B42] Sadler-Smith E. Badger B. (1998). Cognitive style, learning and innovation. *Technol. Anal. Strategic Manag.* 10 247–266. 10.1080/09537329808524314

[B43] Sarfraz M. Qun W. Abdullah M. I. Alvi A. T. (2018a). Employees’ perception of corporate social responsibility impact on employee outcomes: mediating role of organizational justice for small and medium enterprises (SMEs). *Sustainability* 10:2429 10.3390/su10072429

[B44] Sarfraz M. Qun W. Hui L. Abdullah M. I. (2018b). Environmental risk management strategies and the moderating role of corporate social responsibility in project financing decisions. *Sustainability* 10:2771 10.3390/su10082771

[B45] Sarfraz M. Qun W. Sarwar A. Abdullah M. I. Imran M. K. Shafique I. (2019a). Mitigating effect of perceived organizational support on stress in the presence of workplace ostracism in the Pakistani nursing sector. *Psychol. Res. Behav. Manag.* 12:839. 10.2147/PRBM.S210794 31572031 PMC6750711

[B46] Sarfraz M. Qun W. Shah S. G. M. Fareed Z. (2019b). Do hierarchical jumps in CEO succession invigorate innovation? Evidence from the Chinese economy. *Sustainability* 11:2017 10.3390/su11072017

[B47] Shah S. G. M. Tang M. Sarfraz M. Fareed Z. (2019). The aftermath of CEO succession via hierarchical jumps on firm performance and agency cost: evidence from Chinese firms. *Appl. Econ. Lett.* 26 1–5.

[B48] Shepherd D. A. Patzelt H. (2018). “Motivation and entrepreneurial cognition,” in *Entrepreneurial Cognition* (Cham: Palgrave Macmillan), 51–103. 10.1007/978-3-319-71782-1_3

[B49] Sial M. Zheng C. Khuong N. Khan T. Usman M. (2018). Does firm performance influence corporate social responsibility reporting of Chinese listed companies? *Sustainability* 10:2217 10.3390/su10072217

[B50] Staples D. S. Webster J. (2007). Exploring traditional and virtual team members’“best practices”: a social cognitive theory perspective. *Small Group Res.* 38 60–97. 10.1177/1046496406296961

[B51] Tang X. Lin Y. Peng Q. Du J. Chan K. C. (2016). Politically connected directors and firm value: Evidence from forced resignations in China. *North Am. J,. Econ. Finance* 37 148–167. 10.1016/j.najef.2016.04.001

[B52] Torrence B. Connelly S. (2019). Emotion regulation tendencies and leadership performance: an examination of cognitive and behavioral regulation strategies. *Front. Psychol.* 10:1486. 10.3389/fpsyg.2019.01486 31312155 PMC6614202

[B53] Wang L. Liu Q. Hanazaki M. (2019). Corporate board structure and corporate performance: empirical analysis of listed companies in China. *Fudan J. Hum. Soc. Sci.* 12 137–175. 10.1007/s40647-018-0232-0

[B54] Yang D. Wang A. X. Zhou K. Z. Jiang W. (2019). Environmental strategy, institutional force, and innovation capability: a managerial cognition perspective. *J. Bus. Ethics* 159 1147–1161. 10.1007/s10551-018-3830-5

[B55] Yuan Y. Tian G. Lu L. Y. Yu Y. (2019). CEO ability and corporate social responsibility. *J. Bus. Ethics* 157 391–411.

[B56] Zhang H. Ou A. Y. Tsui A. S. Wang H. (2017). CEO humility, narcissism and firm innovation: a paradox perspective on CEO traits. *Leadersh. Q.* 28 585–604. 10.1016/j.leaqua.2017.01.003

[B57] Zhang Y. Qu H. (2016). The impact of CEO succession with gender change on firm performance and successor early departure: Evidence from China’s publicly listed companies in 1997–2010. *Acad. Manag. J.* 59 1845–1868. 10.5465/amj.2014.0176

[B58] Zhao J. Joas R. Abel J. Marques T. Suikkanen J. (2013). Process safety challenges for SMEs in China. *J. Loss Prev. Process Ind.* 26 880–886. 10.1016/j.jlp.2012.09.003

[B59] Zhou C. (2019). Effects of corporate governance on the decision to voluntarily disclose corporate social responsibility reports: evidence from China. *Appl. Econ.* 51 5900–5910. 10.1080/00036846.2019.1631440

[B60] Zhu J. Ye K. Tucker J. W. Chan K. J. C. (2016). Board hierarchy, independent directors, and firm value: evidence from China. *J. Corp. Finance* 41 262–279. 10.1016/j.jcorpfin.2016.09.009

